# Greener and Whiter Analytical Chemistry Using Cyrene as a More Sustainable and Eco-Friendlier Mobile Phase Constituent in Chromatography

**DOI:** 10.3390/ph16101488

**Published:** 2023-10-19

**Authors:** Sami El Deeb, Khalid Abdelsamad, Maria Kristina Parr

**Affiliations:** 1 Institute of Pharmacy, Freie Universität Berlin, Königin-Luise-Str. 2+4, 14195 Berlin, Germany; abdelsamak87@zedat.fu-berlin.de (K.A.); maria.parr@fu-berlin.de (M.K.P.); 2 Institute of Medicinal and Pharmaceutical Chemistry, Technische Universitaet Braunschweig, 38106 Braunschweig, Germany

**Keywords:** green analytical chemistry, white analytical chemistry, sustainability, sustainable solvent, greenness, Cyrene, RGB12, AGREE, RP-HPLC

## Abstract

Cyrene (dihydrolevoglucosenone) was evaluated for the first time as a potential sustainable mobile phase solvent in reversed-phase chromatography. As a benign biodegradable solvent, Cyrene is an attractive replacement to classical non-green organic chromatographic solvents such as acetonitrile and a modifier, co-eluent to known green solvents such as ethanol. Compared to ethanol, Cyrene is less toxic, non-flammable, biobased, biodegradable, and a cheaper solvent. A fire safety spider chart was generated to compare the properties of Cyrene to ethanol and show its superiority as a greener solvent. Cyrene’s behavior, advantages, and drawbacks in reversed-phase chromatography, including the cut-off value of 350 nm, elution power, selectivity, and effect on the column, were investigated using a model drug mixture of moxifloxacin and metronidazole. A monolithic C_18_ (100 × 4.6 mm) column was used as a stationary phase. Different ratios of Cyrene: ethanol with an aqueous portion of sodium acetate buffer mobile phases were tested. A mobile phase consisting of Cyrene: ethanol: 0.1 M sodium acetate buffer pH 4.25 (8:13:79, *v*/*v*/*v*) was selected as the most suitable mobile phase system for separating and simultaneously determining metronidazole and moxifloxacin. The greenness and whiteness of the method were evaluated using the qualitative green assessment tool AGREE and the white analytical chemistry assessment tool RGB12. Further potentials of Cyrene as a solvent or modifier in normal phase chromatography, liquid chromatography–mass spectrometry, and supercritical fluid chromatography are discussed.

## 1. Introduction

Awareness about global climate change has notably increased with the increase in environmental pollution. A global effort is underway to push the environmental protection, economic viability, and social quality pillars of sustainability in various fields. Analytical chemistry plays a dual role in sustainability. On the one hand, it can aid in monitoring soil, air, and water pollutants. On the other hand, it depends on using many (hazardous) chemicals, solvents, and reagents. In 1998, Paul Anastas and John Warner introduced green analytical chemistry (GAC) to reduce the hazardous impact on the environment and the operator [1]. Conducting environmentally benign chemical analysis and thus greening the analytical laboratories is still far from being achieved. Improving analytical chemistry’s impact on the environment and humans still demands further effort towards better chemical and pharmaceutical sustainability. Millions of analytical tests are conducted on a routine daily basis in pharmaceutical and chemical laboratories worldwide. A chromatographic analysis is a big consumer of non-green solvents. Their ecological impact is high, utilizing high-energy resources and producing tons of environmentally hazardous waste [2]. So far, reversed-phase high-performance liquid chromatography (RP-HPLC) is the most widely utilized analytical tool [3]. Nowadays, there is an exponentially increasing trend of applying green analytical chemistry. For example, analytical chemistry regulatory bodies increased sustainability awareness to ensure operators’ safety and environmental preservation. This should consider the steps from sample collection to waste treatment [4].

The Green Chemistry Principle of 12 was formulated by Galuszka et al. to consider different aspects that can make a method greener [5]. Five assessment tools, namely the Eco-Scale [6], the National Environmental method (NEMI) [7,8,9], the Green Analytical Procedure Index (GAPI) [10], the Analytical Method Greenness Score (AMGS) [11], and the Analytical GREEnness Metric Approach and Software (AGREE) [12] were published and used to evaluate the greenness of analytical methods. AGREE is the most useful, as it is the quantitative and more representative assessment of the greenness of analytical procedures. It is open-source software that considers the 12 principles of GAC [12].

White analytical chemistry (WAC) was introduced with a more holistic view to avoid increasing greenness at the expense of method performance and functionality. Thus, it is a more global assessment that considers additional criteria. A balance between the greenness and usefulness of the analytical method should be achieved by considering analytical efficiency and practical and economic aspects, as can be represented by white analytical chemistry. This balance remains a challenge in many cases. Analysts should green the analytical methods without compromising the quality and keep the method useful for its intended application [13].

The RGB12 tool is a good assessment tool to evaluate the whiteness of the analytical method. It is based on analytical efficiency (R), ecological (G), and practical economic efficiency (B), where R and B are not captured by any of the available green assessment tools. Analytical efficiency is represented by validation parameters such as limit of detection (LOD), accuracy, and precision, while practical and economic efficiency (B) express productivity from both aspects. White was then obtained by mixing the three colors, red, green, and blue, to show a score depending on the degree of saturation of each color. A free-access Excel sheet is available to calculate the RGB12 [14]. The RGB model includes the 12 WAC principles, each involving four principles. In practice, R and B should have priority over G to have a method sufficient for the intended application. However, the 12 principles may have different weights and impacts in practice. Coherency and synergy of analytical performance, ecological, and practical aspects form white analytical chemistry. Thus, in analytical chemistry, the aim for sustainable development is, in principle, aiming for the white method.

Various actions can be taken based on the principles of GAC and WAC to make the method greener and whiter. At present, there are several approaches for greening the analytical method via organic solvent replacement, including the use of superheated water [15,16], ethanol [17,18,19,20,21], propylene carbonate [22,23,24], acetone [25,26], natural deep eutectic solvents [27], and micellar [28,29,30,31,32]. These greener solvents have advantages and limitations when applied as mobile phase components in chromatographic applications. Replacing classical mobile phase solvents with eco-friendly, environmentally benign solvents remains an excellent factor in making analytical methods greener and, thus, possibly whiter [3].

**Figure 1:**
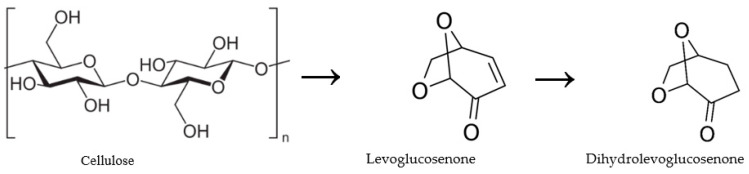
Production scheme of dihydrolevoglucosenone (Cyrene).

Dihydrolevoglucosenone (C_6_H_8_O_3_), also known as Cyrene™, is a bicyclic ketone containing an acetal functional group that is produced in two steps from cellulose biomass through levoglucosenone as presented in Figure 1. Cyrene is biodegradable, non-toxic, and non-mutagenic, in addition to other physicochemical properties of Cyrene, making it a green polar aprotic alternative solvent replacing toxic organic solvents such as dimethyl formamide (DMF), N-methyl pyrrolidone (NMP), N,N-dimethylacetamide (DMAc), and dimethyl sulfoxide (DMSO) in peptide chemistry, material chemistry, and organic synthesis [33,34,35,36,37]. Recently, Cyrene has been used as an alternative sustainable solvent for the preparation of Poly(lactic-co-glycolic acid) nanoparticles [38] and also as an alternative bio-based solvent in solid–liquid extraction [39] and liquid–liquid extraction [40].

**Figure 2:**
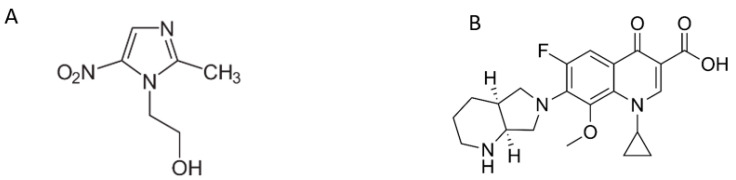
Structural formula of (**A**) metronidazole and (**B**) moxifloxacin.

Metronidazole (1-β-hydroxyethyl-2-methyl-5-nitroimidazole) (MET) is a nitroimidazole antibiotic used in clinical practice since 1959. Metronidazole is active against a wide range of anaerobic bacteria and protozoans [41,42,43]. Moxifloxacin (1-cyclopropyl-7-(2,8-diazo bicyclo [4.3.0.] nonane)-6-fluoro-8-methoxy-1,4-dihydro-4-oxo-3-quinolone carboxylic acid), (MOX) is a fluoroquinolone antibacterial with a methoxy group in the C-8 position. Moxifloxacin has antibacterial activity against a broad spectrum of Gram-positive and Gram-negative organisms [44,45]. The structural formulae of metronidazole and moxifloxacin are shown in Figure 2. Several RP-HPLC methods were reported for the assay of metronidazole and moxifloxacin alone or in combination with other drugs using either acetonitrile or methanol in the mobile phase [46,47,48,49,50]. The simultaneous HPLC determination of metronidazole and moxifloxacin developed by El-Yazbi et al. [51] using acetonitrile was considered a reference method for further comparison.

The main aim of this research was to consider the potential applicability of Cyrene as a mobile phase component in simultaneous chromatographic separation and determination of metronidazole and moxifloxacin. The authors aimed to introduce an innovative RP-HPLC method involving the first-time use of Cyrene as a mobile phase component. Cyrene is investigated as a possible greener chromatographic mobile phase co-eluent together with ethanol. The compatibility of Cyrene as a mobile phase component, UV-absorptivity and cut-off value, eluotropic effect, viscosity, applicability with high flow rates, effect on column performance, selectivity, and other separation aspects is studied. Furthermore, the role of stationary phase morphologies in enhancing green chemistry using monolithic silica columns and the possible application of high flow rates is also considered. Evaluation of the greenness of the developed method using the AGREE metric and the whiteness using the RGB12 tool is conducted as well.

## 2. Results and Discussion

### 2.1. Evaluation of Cyrene as Co-Eluent in the Mobile Phase

Initially, using UV-Vis spectrophotometry, Cyrene’s UV cut-off wavelength was determined to be 350 nm. Therefore, moxifloxacin and metronidazole were selected as model analytes with wide-range absorptivity below and above 350 nm to investigate Cyrene as a mobile phase component. To show the limitations and advantages of Cyrene over classical organic solvents, a comparison of properties to other selected green and non-green reversed-phase solvents has been conducted, as listed in Table 1.

Cyrene has one main drawback that limits its use in chromatography. This drawback is the high cut-off value of 350 nm, which covers most of the UV region and is above the maximum absorbance wavelength of most pharmaceuticals. This cut-off value is significantly higher than that of acetonitrile and methanol and also higher than that of ethanol. Therefore, the UV absorptivity of Cyrene is a significant obstacle. Our work showed successful detection for compounds eluted using Cyrene in the mobile phase at a detection wavelength of 350 nm. Detectability with a noisy baseline was also possible below the cut-off value of Cyrene at 320 nm. However, one should consider that Cyrene is not fully compatible with the UV/Vis detector. It would still work well with compounds having strong UV chromophores as dyes and aromatic hydrocarbons, making it easily possible to carry out green HPLC analysis in industries handling such substances or more suitable to environmental areas or hazardous, restricted areas, thus enhancing a benign analysis technology that might be possible in offices in the future. Even though UV/Vis detection is the most common primary detection mode in HPLC, Cyrene is expected to work better without this limitation using other detection techniques like electrochemical detection. Most attractive is the try of Cyrene with MS detection as a solvent for LC-MS and LC-MS/MS to ensure that it does not pose a problem if the mobile phase contains certain Cyrene and to see its volatility, effect on ionization efficiency and general compatibility with different ion sources. The authors are currently investigating the compatibility with the MS detector. Cyrene will also be investigated as a possible co-eluent in supercritical fluid chromatography.

As mentioned in Table 1, Cyrene has a high flash point of 108 °C, which is higher than all the other solvents compared in the table except propylene carbonate. In principle, a flash point higher than 60 °C is considered safe. In contrast to Cyrene, ethanol has a very low flash point of 9.7 °C and is considered a flammable solvent. The density of Cyrene is higher than other solvents; however, using a monolithic column with a bimodal pore structure and high porosity reduces the generated backpressure even at a higher flow rate than 1 mL/min, as shown in Section 2.3. Cyrene has a higher value of the Kamlet–Taft π* solvent parameter than the other solvents mentioned in Table 1, indicating higher polarity and, thus, weaker elution power in RP-HPLC. It is worth mentioning here that increasing the column temperature would increase the elution power of Cyrene, as described in Section 2.3.

**Figure 3:**
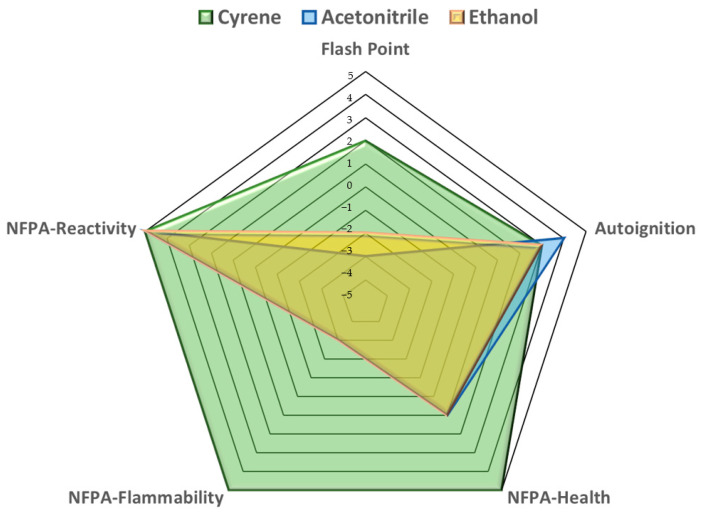
The spider chart generated based on the data presented in Table 2 reveals that Cyrene significantly outperforms ethanol in fire safety.

The fire safety of Cyrene was compared to that of acetonitrile and ethanol, as shown in Table 2. A fire safety chart has also been generated to show Cyrene’s superiority as a greener solvent over ethanol, as presented in Figure 3. No flammability or significant health and reactivity hazards are associated with Cyrene.

**Figure 4:**
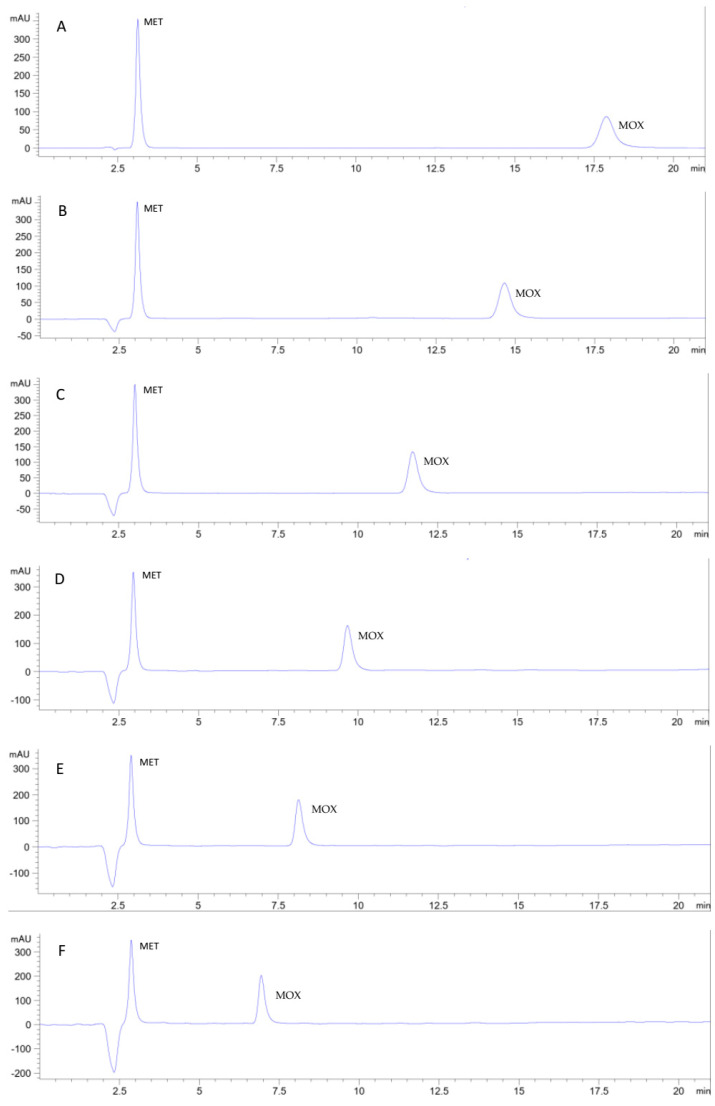
Chromatograms showing the separation of metronidazole and moxifloxacin on a monolithic C18 (100 × 4.6 mm) column at a flow rate of 1 mL/min and detected at 350 nm. The two drugs were eluted using different mobile phase compositions: (**A**): ethanol: 0.1 M sodium acetate buffer pH 4.25 (13:87, *v*/*v*), (**B**): Cyrene:ethanol: 0.1 M sodium acetate buffer pH 4.25 (2:13:85, *v*/*v*), (**C**): Cyrene:ethanol: 0.1 M sodium acetate buffer pH 4.25 (4:13:83, *v*/*v*/*v*), (**D**): Cyrene:ethanol: 0.1 M sodium acetate buffer pH 4.25 (6:13:81, *v*/*v*/*v*), (**E**): Cyrene:ethanol: 0.1 M sodium acetate buffer pH 4.25 (8:13:79, *v*/*v*/*v*), and (**F**): Cyrene:ethanol: 0.1 M sodium acetate buffer pH 4.25 (10:13:77, *v*/*v*/*v*).

To study the potential use of Cyrene as a mobile phase component in RP-HPLC, a method using an ethanol and sodium acetate buffer was first developed as a green ethanol-based reference method (Figure 4A) in replacement of the reported acetonitrile-based non-green method [51] for the simultaneous determination of the two analytes. Further reduction of the analysis time of the developed ethanol-based method may be possible by increasing the percentage of ethanol in the mobile phase. However, we aimed to reduce the consumption of ethanol and alternatively reduce the analysis time, including the greener solvent Cyrene as a co-eluent in the mobile phase.

Cyrene was added as a co-eluent to the ethanol: 0.1 M sodium acetate buffer pH 4.25 (13:87, *v*/*v*) mobile phase, and the effects on elution, detectability, selectivity, and backpressure were monitored and reported. A successive increase in Cyrene percentage in the mobile phase with a 2% step substitution of a buffer was found to increase the elution power of the mobile phase in synergy with ethanol as two organic eluents and thus reduce the total analysis time. No change in selectivity was observed. The effect on detectability was reported by monitoring the absorbance in the 200–400 nm range. The two analytes were found to be detectable at 320 nm (a value below the cut-off value of Cyrene) but with higher noise. The best detectability was obtained at 350 nm. A Cyrene mobile phase percentage of up to 8% reduced the run time while preserving a smooth baseline with good detectability. At this percentage, the increased backpressure due to the higher density of Cyrene was tolerable. A proportion of 10% Cyrene in the mobile phase resulted in an even shorter analysis time. However, the peak of metronidazole was eluted very close to the solvent peak with a relatively noisy baseline. Therefore, the mobile phase consisting of Cyrene: ethanol: 0.1 M sodium acetate buffer pH 4.25 (8:13:79, *v*/*v*/*v*) was selected as the best suitable mobile phase system for separating and simultaneously determining metronidazole and moxifloxacin. The developed method was validated according to ICH guidelines [54]. Important validation parameters are summarized in Table 3. The method showed linearity over the studied ranges with good accuracy and precision values. Detection and quantitation limits fit the intended purpose. Representative chromatograms for metronidazole and moxifloxacin elution using different percentages of Cyrene in the mobile phase are shown in Figure 4.

**Figure 5:**
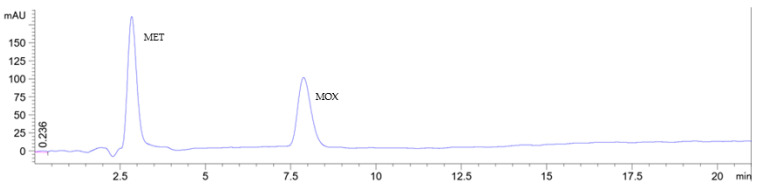
Chromatograms showing the separation of metronidazole and moxifloxacin on a monolithic C18 (100 × 4.6 mm) column at a flow rate of 1 mL/min and detected at 350 nm. The two drugs were eluted using a Cyrene: ethanol: 0.1 M sodium acetate buffer pH 4.25 (8:13:79, *v*/*v*/*v*), which was also used as a sample solvent.

For the selected Cyrene-based RP-HPLC method, the observed negative solvent peak has been avoided by applying the mobile phase as the sample solvent, as shown in Figure 5.

Cyrene did not negatively impact column performance when a pre- and post-run with ethanol: 0.1 M sodium acetate buffer pH 4.25 mobile phases were compared for the elution of metronidazole and moxifloxacin. No significant changes in retention times (coefficient of variation percent (CV%) of metronidazole 0.19 and moxifloxacin 0.50), peak areas (CV% of metronidazole 1.61 and moxifloxacin 6.27), and theoretical plates (CV% of metronidazole 0.61 and moxifloxacin 2.86) were obtained. However, the long-term effect on column half-life needs further investigation. Cyrene has a higher density than all common green and non-green RP-HPLC solvents. However, the used mobile phases consisting of a Cyrene: ethanol: sodium acetate buffer in different ratios generated acceptable backpressures, as shown in Table 4, which would work with all classical conventional columns. Using monolithic silica columns with a bimodal pore structure and high porosity makes this issue less significant. Cyrene shows a weak eluotropic strength and, thus, weaker elution power than ethanol in reversed-phase chromatography in agreement with the Kamlet–Taft polarity parameter value. The unique eluotropic strength of Cyrene could be further investigated and defined using a larger number of compounds or model drugs with high chemical diversity. Cyrene may also be applied as a co-eluent/modifier to fine-tune the elution and improve the separation of a complex mixture. It is worth emphasizing that because ethanol has stronger eluotropic strength than methanol and acetonitrile, a mixture of ethanol and Cyrene may produce an equivalent eluotropic strength compared to methanol or acetonitrile portions in mobile phases. This may result in easier method transfer from methanol or acetonitrile to Cyrene/ethanol and, thus, easier switching to greener methods. Cyrene showed a similar selectivity to other classical solvents such as acetonitrile, methanol, and the green solvent ethanol in the RP-LC. Therefore, it does not impact the elution order when substituting other classically used organic solvents in the mobile phase to make the method greener.

### 2.2. Greenness and Whiteness Assessments of the Methods

**Figure 6:**
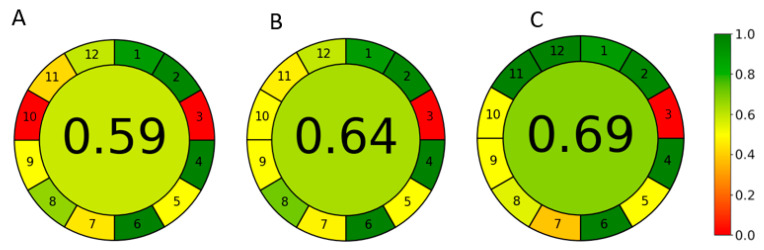
Greenness evaluation of (**A**) the reported acetonitrile-based non-green method, (**B**) the developed green ethanol-based reference method, and (**C**) the developed greener Cyrene/ethanol-based method using AGREE tool.

The greenness of the three methods with the elution conditions listed in Table 5 was evaluated and compared using the quantitative greenness assessment tool AGREE. Greenness profiles are presented in Figure 6. Results showed that the developed Cyrene/ethanol-based method has superior greenness profiles.

**Figure 7:**
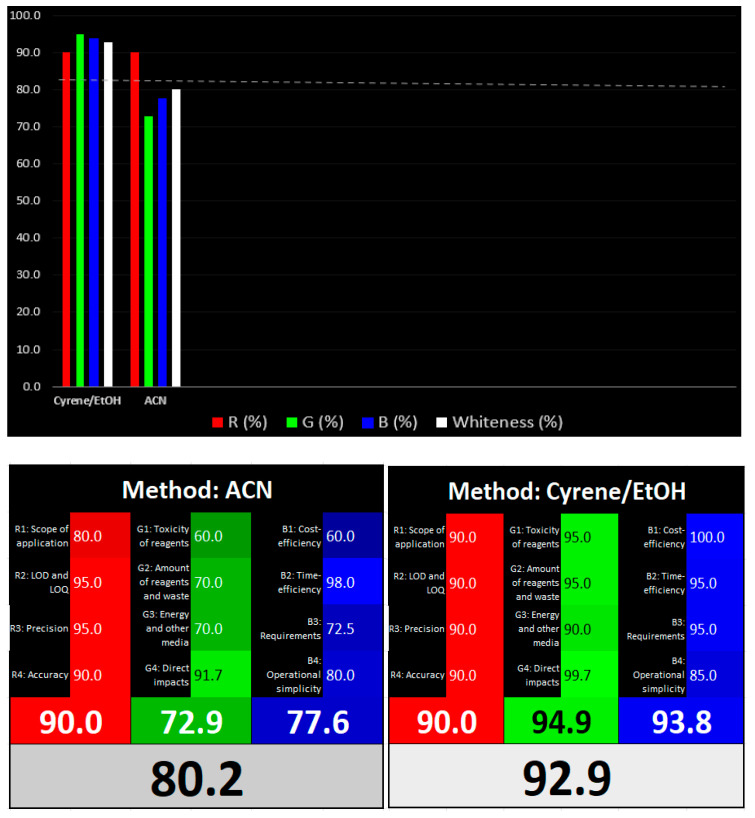
Evaluation of the whiteness of the reference ACN and the developed Cyrene: EtOH methods according to the RGB12 tool.

Furthermore, the whiteness of the Cyrene-based method was evaluated and compared with the reference acetonitrile method using the RGB12 tool. The results presented in Figure 7 show that the Cyrene-based method is regarded as a whiter analytical method mainly because of the better green and blue components without compromising the red component of the evaluation matrix.

### 2.3. Potential of Heated/Superheated Cyrene Chromatography vs. Heated/Superheated Water Chromatography

**Figure 8:**
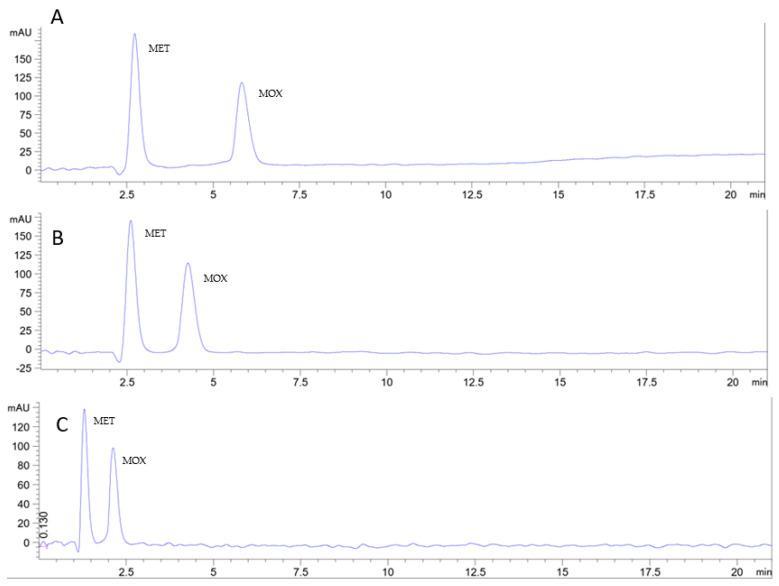
Chromatograms show the separation of metronidazole and moxifloxacin on a monolithic C18 (100 × 4.6 mm) column. The two drugs were eluted using a Cyrene: ethanol: 0.1 M sodium acetate buffer pH 4.25 (8:13:79, *v*/*v*/*v*), also used as a sample solvent. The two drugs were detected at 350 nm. (**A**) Column temperature 35 °C and flow rate 1 mL/min, (**B**) column temperature 50 °C and flow rate 1 mL/min, and (**C**) column temperature 50 °C and flow rate 2 mL/min.

The best selected successfully developed Cyrene-containing RP-HPLC method at ambient temperature and a flow rate of 1 mL/min for the elution of the metronidazole moxifloxacin mixture with the mobile phase Cyrene: ethanol: sodium acetate buffer pH 4.25 (8:13:79, *v*/*v*/*v*) was then tested at higher temperatures of 35 °C and 50 °C. Increasing the temperature from ambient 25 °C to 35 °C and then to 50 °C at the flow rate of 1 mL/min reduced the analysis time and the backpressure [55,56,57]. Chromatograms showing elution at high temperatures are presented in Figure 8A,B. Increasing the temperature is supposed to decrease the dielectric constant and strengthen the elution power while decreasing the viscosity and, thus, the generated backpressure. Keeping in mind the higher energy consumption associated with higher column temperature will negatively impact the greenness and, thus, the whiteness of the method, conducting the chromatographic analysis at an increased flow rate of 2 mL/min and a high column temperature of 50 °C resulted in a significant reduction in the retention time with a relatively noisy baseline but acceptable backpressure of 130 bar. A chromatogram showing elution at higher temperatures of 50 °C and a higher flow rate of 2 mL/min is presented in Figure 8C.

The eluotropic strength of Cyrene is still stronger than water and increases at higher column temperature and eventually at higher mobile phase temperature if a mobile phase oven or mobile phase water bath as a mobile phase preheater is applied using the conventional HPLC system. Increasing the column temperature from ambient to 50 °C without approaching the maximum allowed monolithic silica column temperature of 60 °C would enhance the elution power of Cyrene by decreasing the dielectric constant and viscosity, which also decreases the generated backpressure. Thus, a promising application of Cyrene in chromatography may be a kind of what could be called “heated Cyrene chromatography” (with or without water as other mobile phase constituents) inconsistent with what is known as superheated water chromatography. The limitations of facing insoluble hydrophobic compounds when applying heated water chromatography do not apply in heated Cyrene or heated Cyrene water chromatography because of the good solving power of Cyrene. It is worth noting that high-temperature chromatography would be more successful using temperature-resistant stationary phase columns such as polystyrene divinyl benzene and zirconia particles with polybutadiene. Our initial investigations showed improved elution strength and lower generated backpressure, indicating a potentially successful, promising elution principle. This may even be more successful for normal phase chromatography for eluting polar analytes. The higher used temperature and thus energy and the need for additional equipment to heat the mobile phase will negatively impact GAC and WAC.

## 3. Materials and Methods

### 3.1. List of Chemicals

The reference material of metronidazole was obtained from Caelo (Hilden, Germany), and moxifloxacin was obtained from Sigma–Aldrich Chemie GmbH (Darmstadt, Germany). Ethanol > 99.7% HPLC grade was obtained from VWR International S.A.S. (Rosny-sous-Bois, France). Glacial acetic acid for HPLC was obtained from Applichem (Darmstadt, Germany). Cyrene™ and sodium acetate were obtained from Merck (Darmstadt, Germany). Hydrochloric acid analytical grade was obtained from Fisher Scientific (Loughborough, UK).

### 3.2. Buffer and Sample Preparation

Sodium acetate buffer 0.1 M pH 4.25 was prepared by adding 5.772 g of sodium acetate and 1.778 g of acetic acid to 800 mL bidistilled water. The pH was adjusted to 4.25 by adding 10 N HCl, and the volume was completed to 1 L by bidistilled water. Stock solutions of 500 µg/mL of moxifloxacin and 1000 µg/mL of metronidazole were made for the preparation of calibrants and quality control samples.

### 3.3. HPLC Analysis

An Agilent 1260 (Agilent Technologies GmbH, Waldbronn, Germany) equipment with a quaternary pump (G1311B), autosampler (G1329B), and diode array detector (G1315D) was used. The Chromolith Performance RP-18e (100 × 4.6 mm) column (Merck, Darmstadt, Germany) was used.

### 3.4. Method Validation

Stock solutions of metronidazole and moxifloxacin were prepared in bidistilled water at concentrations of 1000 and 500 µg/mL, respectively. The solutions were then used to prepare standards for the validation study. For establishing linearity, calibration curves were generated by plotting peak areas of analytes against their corresponding concentrations using seven different standard concentrations0.3, 1, 5, 10, 20, 40, and 80 µg/mL) for metronidazole and five different standard concentrations (10, 20, 40, 60, and 80 µg/mL) for moxifloxacin. For accuracy testing, three quality control (QC) standards at low (LQC), medium (MQC), and high (HQC) concentration levels within the linear range using 5, 10, and 40 µg/mL for metronidazole and 10, 40, and 60 µg/mL for moxifloxacin were investigated. QC standards were then used to establish the precision of the proposed methodology.

LOQ was calculated based on the standard deviation of the response and the slope using the equation LOQ = 10 σ/S, where σ is the standard deviation of the response, and S is the slope of the calibration curve. The LOD was calculated based on the standard deviation of the responses and the slope using the equation LOD = 3.3 σ/S.

## 4. Conclusions

In this study, Cyrene was applied for the first time as a constituent of the mobile phase in chromatography. Cyrene showed strong potential as a chromatographic organic modifier component in the mobile phase. The present study developed and validated the first RP-HPLC method using Cyrene as a co-eluent with ethanol in the mobile phase. Using Cyrene in the mobile phase resulted in scaling down the amount of the green ethanol and substituting it with Cyrene as a greener solvent. The two green solvents, Cyrene and ethanol, were used successfully to carry out the chromatographic separation.

Cyrene has a higher viscosity than other classical mobile phase organic solvents and limited solubility with water and an aqueous buffer at high concentrations. The high backpressure might result in greater wear on instrumentation. However, practical experiments showed that the generated backpressure with the low needed percentage of Cyrene in the mobile phase of less than 10% is below 100 bar despite higher viscosity, which will be affordable even with classical conventional columns. It is worth mentioning that Cyrene could also perform better with ultra-high-performance liquid chromatographic equipment where pressure tolerance on instrumentation is higher. However, the higher viscosity of Cyrene and, thus, the higher developed backpressure would not be a problem when using monolithic silica columns as a stationary phase because of the high permeability and lower developed backpressure. No significant change in monolithic silica column performance was noted when regenerating ethanol/buffer methods before and after the use of Cyrene on a monolithic silica column, and good precision has been reported. However, the long-term effect of Cyrene on column life is under investigation.

Cyrene also greens the sample by substituting other toxic or less green organic sample solvents if required to solubilize non-water-soluble analytes. In large-scale industries, this may also reduce risk hazards. Using Cyrene as a sample solvent component instead of, e.g., methanol enhances the greenness and, thus, the whiteness of the analytical method. It is worth noting that Cyrene has shown good capacity to solubilize organic compounds. From the economic aspect of sustainability, Cyrene is cheaper than HPLC-grade acetonitrile and much cheaper than HPLC-grade ethanol. It is not as toxic as both methanol and acetonitrile or as toxic as ethanol and not as flammable as the three other mentioned solvents but rather an eco-friendly bio-based biodegradable solvent that would even reduce and avoid costs required for waste treatment. Using Cyrene as a green, benign, bio-based, and biodegradable solvent also reduces expensive and time-consuming waste treatment, thus potentially decreasing the cost of analysis. Therefore, the developed method will be rendered more cost-effective from an economic standpoint with green eluents and green effluents. Cyrene has a high boiling point and flashpoint temperature, thus reducing the fire possibilities in laboratories and enhancing the analytical method’s greenness by reducing the use of flammable ethanol and substituting it with non-flammable. Compared to the reported non-green acetonitrile and the developed ethanol-based green method, the developed Cyrene-based method showed superior greenness and whiteness scores. The boiling point affects pump performance and safety. Thus, higher boiling solvents such as Cyrene, which has a boiling point of 227 °C, are usually preferred.

Results showed the potential for Cyrene to replace hazardous non-green organic solvents in combination with ethanol and possibly alone if high temperature is also considered or other detection modes are applied. Method accuracy and precision were maintained, while the method efficiency was not compromised. Results showed similar redness to the acetonitrile and Cyane-based methods, and the obtained LOQ is still appropriate and sufficient. Therefore, the main added effect on the RGB12 tool is the significant increase in the greenness and blueness aspects due to Cyrene’s use. Even though the LOD and LOQ are somewhat higher, giving a good score is fair because they are sufficient for the intended application. Good accuracy percentages were obtained for metronidazole in the range of 98.4–105.6% and for moxifloxacin in the range of 96.7–104.7%. The method also showed good precision with RSD% < 2. The critical factor remains the sufficiency of the method to fit its intended purpose. Thus, a good whiteness percentage was achieved.

## Figures and Tables

**Table 1:** Physicochemical properties of Cyrene versus selected green and non-green RP-HPLC solvents *.

Solvent	UV Cut-Off Value (nm)	Water Solubility	Density (g/cm^3^) at 20 °C	Polarity Parameter Kamlet–Taft π*	Partition Coefficient N-Octanol/Water (Log Value)	Boiling Point °C	Flash Point °C at 1.013 hPa (c.c.)
Acetonitrile	190	Miscible in any proportion	0.7822	0.75	−0.34	82	2
Methanol	205	1000 g/L at 20 °C—completely miscible	0.7913	0.61	−0.77	64.7	12
Ethanol	210	≥1000 g/L at 20 °C	0.81	0.54	−0.31	78	9.7
Water	190	Not applied	0.9982	1.2	Not applied	100	Not applied
Propylene carbonate	220	175 g/L at 25 °C	1.2047	0.9	−0.41	240	132
Acetone	330	Miscible in any proportion	0.79	0.71	−0.23	56.05	−18
Cyrene	350	ca.52.6 g/L at 20 °C	1.25	0.93	−1.52	227	108

* Data were collected from solvents, safety data sheets [52], and references [8,9,33,53]. The UV cut-off value was determined at the laboratories of FU Berlin.

**Table 2:** Fire safety criteria for acetonitrile, ethanol, and Cyrene *.

Solvent Evaluation Criteria		Flash Point	Autoignition	NFPA Health	NFPA Flammability	NFPA Reactivity
Acetonitrile	Value or Rating	2 °C	524 °C	2	3	0
	Score	−3	4	1	−3	5
Ethanol	Value or Rating	12 °C	455 °C	2	3	0
	Score	−2	3	1	−3	5
Cyrene	Value or Rating	108 °C	296 °C	0	0	0
	Score	2	3	5	5	5

* Data were obtained from solvent safety data sheets [52].

**Table 3:** Summary of important validation parameters for the developed Cyrene/ethanol-based method.

Parameter	MET	MOX
Linearity (R^2^)	0.999	0.9994
Linearity Range (µg/mL)	0.3–80	10–80
LOD (µg/mL)	0.1	4
LOQ (µg/mL)	0.3	8
Accuracy (µg/mL)	98.4–105.6%	96.7–104.7%
Precision RSD%		
LQC	1.4%	1.9%
MQC	1.2%	1.6%
HQC	0.8%	1.6%

**Table 4:** Generated backpressures for the used mobile phase compositions on a monolithic C18 (100 × 4.6 mm) column.

Mobile Phase Composition	Generated Backpressure in Bar
Ethanol: 0.1 M sodium acetate buffer pH 4.25 (13:87, *v*/*v*)	71
Cyrene: 0.1 M ethanol: sodium acetate buffer pH 4.25 (2:13:85, *v*/*v*/*v*)	75
Cyrene: 0.1 M ethanol: sodium acetate buffer pH 4.25 (4:13:83, *v*/*v*/*v*)	80
Cyrene: 0.1 M ethanol: sodium acetate buffer pH 4.25 (6:13:81, *v*/*v*/*v*)	85
Cyrene:ethanol: 0.1 M sodium acetate buffer pH 4.25 (8:13:79, *v*/*v*/*v*)	91
Cyrene:ethanol: 0.1 M sodium acetate buffer pH 4.25 (10:13:77, *v*/*v*/*v*)	98

**Table 5:** Elution conditions of the reported and developed methods for the simultaneous determination of metronidazole and moxifloxacin.

Method		Elution Conditions	Reference
A	Reported acetonitrile-based non-green method	HPLC-DAD using RP-C18 column. Isocratic elution using ACN and phosphate buffer (30:70, *v*/*v*) as mobile phase on a Zorbax Eclipse Plus C18 column	[51]
B	Developed green ethanol-based reference method	HPLC-DAD using monolithic C18 column. Isocratic elution using ethanol and 0.1 M sodium acetate buffer pH 4.25 (13:87, *v*/*v*) as mobile phase	This work
C	Develop a greener Cyrene/ethanol-based method.	HPLC-DAD using monolithic C18 column. Isocratic elution using Cyrene: ethanol and0.1 M sodium acetate buffer pH 4.25 (8:13:79, *v*/*v*/*v*) as mobile phase	This work

## Data Availability

Data is contained within the article.
